# Ward Atmosphere and Patient Satisfaction in Psychiatric Hospitals With Different Ward Settings and Door Policies. Results From a Mixed Methods Study

**DOI:** 10.3389/fpsyt.2019.00576

**Published:** 2019-08-30

**Authors:** Simone Agnes Efkemann, Johannes Bernard, Janice Kalagi, Ina Otte, Bianca Ueberberg, Hans-Jörg Assion, Swantje Zeiß, Peter W. Nyhuis, Jochen Vollmann, Georg Juckel, Jakov Gather

**Affiliations:** ^1^Department of Psychiatry, Psychotherapy and Preventive Medicine, LWL University Hospital, Ruhr University Bochum, Bochum, Germany; ^2^Institute for Medical Ethics and History of Medicine, Ruhr University Bochum, Bochum, Germany; ^3^LWL-Klinik Dortmund, Psychiatrie, Psychotherapie, Psychosomatische Medizin, Rehabilitation, Dortmund, Germany; ^4^Klinik für Psychiatrie, Psychotherapie und Psychosomatik, St. Marien Hospital Eickel, Herne, Germany

**Keywords:** open-door policies, acute psychiatry, qualitative-empirical interviews, mixed methods, EssenCES, ZUF-8

## Abstract

**Background:** Open-door policies in psychiatry are discussed as a means to improve the treatment of involuntarily committed patients in various aspects. Current research on open-door policies focuses mainly on objective effects, such as the number of coercive interventions or serious incidents. The aim of the present study was to investigate more subjective perceptions of different psychiatric inpatient settings with different door policies by analyzing ward atmosphere and patient satisfaction.

**Methods:** Quantitative data on the ward atmosphere using the Essen Climate Evaluation Scale (EssenCES) and on patient satisfaction (ZUF-8) were obtained from involuntarily committed patients (*n* = 81) in three psychiatric hospitals with different ward settings and door policies (open, facultative locked, locked). Furthermore, qualitative interviews with each of 15 patients, nurses, and psychiatrists were conducted in one psychiatric hospital with a facultative locked ward comparing treatment in an open vs. a locked setting.

**Results:** Involuntarily committed patients rated the EssenCES’ subscale “Experienced Safety” higher in an open setting compared with a facultative locked and a locked setting. The subscale “Therapeutic Hold” was rated higher in an open setting than a locked setting. Regarding the safety experienced from a mental health professionals’ perspective, the qualitative interviews further revealed advantages and disadvantages of door locking in specific situations, such as short-term de-escalation vs. increased tension. Patient satisfaction did not differ between the hospitals but correlated weakly with the EssenCES’ subscale “Therapeutic Hold.”

**Conclusion:** Important aspects of the ward atmosphere seem to be improved in an open vs. a locked setting, whereas patient satisfaction does not seem to be influenced by the door status in the specific population of patients under involuntary commitment. The ward atmosphere turned out to be more sensitive to differences between psychiatric inpatient settings with different door policies. It can contribute to a broader assessment by including subjective perceptions by those who are affected directly by involuntary commitments. Regarding patient satisfaction under involuntary commitment, further research is needed to clarify both the relevance of the concept and its appropriate measurement.

## Background

The issue of coercion in psychiatry has been strongly debated in the past few years (1–3). Applying coercion requires an ethical and legal justification. Coercive measures and involuntary commitments in psychiatry in many European jurisdictions are legally based on the criterion of acute danger to self or to others and aim to avert harm for the involuntarily committed person or for third parties (4, 5). To reach this goal, it is a common practice to treat involuntarily committed patients in psychiatry on permanently locked wards (6).

In recent years, however, open-door policies, i.e., the reference of involuntarily committed patients to open instead of locked wards, have been intensively discussed as an alternative to the tradition of locked wards (7, 8). The major aims of open-door policies are to reduce the use of coercion and to strengthen patients’ autonomy (9). Several psychiatric hospitals in Germany are trying to implement the concept, not least because of legal changes in some federal state mental health laws which state explicitly that legal commitments should be realized in an open setting, as far as possible. The concrete implementation of open-door policies ranges from the facultative opening of the doors of some wards for intermittent periods of time to permanently opening all wards of a psychiatric hospital (10).

A successful implementation of open-door policies requires several conceptual and organizational changes which, among others, include a strengthening of the therapeutic relationships, new security concepts and a further development of the therapeutic milieu (11–13). Burns (14) also argues for an enhancement of therapeutic engagement instead of emphasizing aspects of compulsion and control by locking the doors. Such claims have implications for research on open-door policies. Up to now, most studies have analyzed the effects of open wards regarding mainly objective security and risk management aspects, such as coercive measures, absconding, suicide attempts, or violence (7, 8, 15–18). This can lead to a disregard of other indicators, which go beyond objective security aspects and take into account the subjective perception of those who are directly affected by the involuntary commitment.

Two such more subjective indicators are ward atmosphere and patient satisfaction. Both concepts are sometimes discussed and applied together, but the character and the direction of the relationship between ward atmosphere and patient satisfaction remain unclear (19, 20).

Ward atmosphere was developed specifically for psychiatric settings and used several decades ago in the context of treatment outcome measurements (21, 22). In recent years, it has been discussed increasingly in the context of violence and aggression (23–25). Some complex interventions, such as the so-called engagement model, aim to reduce coercion by improving the ward atmosphere, i.e., by creating an “atmosphere of hospitability and warmth” and, thereby, strengthening the therapeutic alliance (26–28). There are also some studies which investigated ward atmosphere in relation to certain ward characteristics: Schalast and Sieß (29) examined the ward atmosphere experienced from patients and mental health professionals’ perspectives in different psychiatric settings, with slightly different results for both groups. Among others, they showed that some aspects of ward atmosphere were rated lower on locked than open wards. Blaesi et al. (30) investigated ward atmosphere explicitly in the context of open-door policies, complemented by a four-year follow up after the opening of wards (31). They concluded that door opening influences ward atmosphere positively.

Patient satisfaction is a rather unspecific indicator which is commonly applied in the evaluation of health care interventions in general (32, 33). In the past decades, several scales have been developed and used to assess patient satisfaction in the specific context of mental health care (34, 35). There is evidence that patients who are more satisfied with the treatment received tend to be more adherent to therapies and profit more from them (36). Regarding involuntarily committed patients, this implies that an improvement of patient satisfaction might contribute to a reduction of involuntary interventions by enhancing treatment adherence and outcomes and preventing treatment refusals. Furthermore, Woodward et al. (37) point out that patient satisfaction is influenced, in addition to patient-related factors, by setting characteristics. This includes studies indicating that admission status, door status, and experience of coercion can have an impact on patient satisfaction (37–40). This leads to the question whether a psychiatric inpatient setting with an open-door policy can improve the satisfaction of involuntarily committed patients.

To the best of our knowledge, there have been no combined assessments of ward atmosphere and patient satisfaction in the specific context of different psychiatric inpatient settings with different door policies so far. Additionally, we are not aware of any studies concerning ward atmosphere and patient satisfaction which examined involuntarily committed patients directly by including them both in a quantitative and qualitative study. Therefore, our present study, first, aimed to investigate the differences in ward atmosphere and patient satisfaction in three psychiatric hospitals with different ward settings and door policies from the perspective of involuntarily committed patients, using standardized questionnaires. Second, we aimed to qualitatively assess patients and mental health professionals’ experiences and attitudes toward open and locked wards in a facultative locked hospital. In this context, we intended to gain further insights into the relationships between different door policies, ward atmosphere, and patient satisfaction.

## Methods

The data presented in this paper are part of a larger mixed methods study consisting of a quantitative and a qualitative subproject. The study was approved by the Research Ethics Committee of the Medical Faculty of the Ruhr University Bochum (Reg. No. 15-5452).

The quantitative subproject included a standardized documentation and assessment of all involuntary commitments over a period of 6 months, between September 2016 and March 2017, in psychiatric hospitals with different ward settings and door policies. The documentation and assessment started with the first and ended with the last day of the involuntary commitment. Five hospitals were initially recruited for the study, but only four hospitals finally participated in the study. The additional assessment of ward atmosphere and patient satisfaction using standardized questionnaires was conducted only in three hospitals, so only results from the latter will be presented in this paper.

### Hospital Description

All institutions participating were acute psychiatric hospitals located in the Ruhr Area in North-Rhine Westphalia, a federal state in Western Germany. They all had the obligation to admit involuntarily committed patient resident in a hospital’s defined catchment area. As the sizes of these catchment areas were different, the hospitals participating differed in the total number of beds (hospital 1: 464, hospital 2: 137, hospital 3: 159). The three hospitals pursued different strategies to manage involuntary commitments including different door policies. No hospital participating made major conceptual changes during the period assessed.

In the first hospital, all involuntarily committed patients were referred to admission wards which were permanently locked. As a rule, these patients were not transferred to open wards until the end of their involuntary commitment. This means that all involuntarily committed patients included in that hospital stayed on permanently locked wards during the whole time of the assessment. Therefore, we refer to this hospital as “locked.” The second hospital (referred to as “facultative locked”) distributed all patients, including those who were involuntarily committed, to specialized wards according to their diagnosis. With this strategy, the hospital aimed to treat all patients on open wards. However, the mental health professionals could apply restrictive strategies in specific situations to prevent patients from absconding, including temporal door locking of a smaller division (10 of 32 beds) of the ward for patients with psychotic disorders. In the third hospital, all wards were permanently open. Acutely ill and involuntarily committed patients were evenly distributed over all non-diagnosis-specific wards with their doors never locked. Therefore, we labeled this hospital as “open.”

### Standardized Questionnaires

All involuntarily committed patients with preserved mental capacity were asked to fill out standardized questionnaires. Among others, the ward atmosphere was assessed with the Essen Climate Evaluation Scale (EssenCES) (41) and the patient satisfaction with the German adaptation of the Client Satisfaction Questionnaire (CSQ), the so-called ZUF-8 (42). The EssenCES consists of three different subscales: “Patients’ Cohesion and Mutual Support,” referring to the cohesion among the patients themselves, “Therapeutic Hold,” focusing on the therapeutic relationship between patients and mental health professionals and “Experienced Safety (vs. threat of aggression and violence)” (41). It was originally developed in the context of forensic psychiatry but has also been shown to be a valid tool to assess ward atmosphere in general psychiatry (43). Each subscale consists of five items, coded from 0 to 4, thus, a maximum of 20 can be achieved for each scale with higher values representing a better rating. The ZUF-8 is an eight-item questionnaire on satisfaction with the inpatient care. Each item is coded from 1 (maximally negative) to 4 (maximally positive), therefore, an overall maximum of 32 can be reached with higher values representing a higher satisfaction (42). Furthermore, the severity of illness and level of psychosocial functioning were assessed by the treating psychiatrists using the Clinical Global Impressions (CGI) Scale (44) and the Psychosocial Performance Scale (PSP) (45).

The assessments described in this section were conducted not earlier than 72 hours before the end of the involuntary commitment. Data were analyzed using SPSS 25, calculating an ANOVA for each value. Pair-wise comparison was conducted with *post hoc* tests using Bonferroni. Subsamples were compared by using ANOVA for parametric measures and the Kruskal Wallis and the Chi² test for categorical measures, such as gender and diagnoses. A total of *n* = 81 (10.6%) gave their informed consent to participate in the additional survey and filled out the questionnaires (**Table 1**). When comparing the response rates between the three hospitals, it is striking that the rate is much lower in the first hospital. However, despite the differing size of the hospitals the characteristics of participating patients, such as gender or age (**Table 2**), of the subsample from the locked hospital did not differ from the total sample. Only a slightly higher number of patients being committed more than once during the period assessed could be observed. Additionally, sample sizes were sufficiently equal and large to conduct statistical analyses on these data. Furthermore, the subsample of patients participating did not differ significantly in these characteristics from the overall sample of involuntarily committed patients (which is not presented in detail in this paper), indicating that there was no bias between participating and nonparticipating patients.

**Table 1:** Involuntary commitments and response rate per hospital.

	Hospital 1 (locked)			Hospital 2 (facultative locked)			Hospital 3 (open)	
	***n***	***%***		***n***	***%***		***n***	***%***
Number of involuntary commitments	754			119			29	
Number of involuntarily committed patients	632			106			28	
Response rate for questionnaires	23	3.6		44	41.5		14	50.0

**Table 2:** Sample characteristics in total and per hospital.

	Total			Hospital 1 (locked)			Hospital 2 (facultative locked)			Hospital 3 (open)	
	***M/n***	***SD/%***		***M/n***	***SD/%***		***M/n***	***SD/%***		***M/n***	***SD/%***
Gender (male)	44	54.3		12	52.2		24	54.5		8	57.1
Age	41.9	14.5		42.5	12.4		41.8	16.1		41.2	13.1
Multiply committed during study period (yes)	7	8.6		3	13.0		4	9.1		0	0
Previous inpatient stays	3.1	4.7		2.5	3.4		2.9	5.0		4.3	5.7
Previous involuntary commitments	0.7	1.2		1.0	1.4		0.6	1.1		0.7	1.2
Diagnosis											
Substance disorders (ICD-10 F1)	11	13.8		3	13.6		6	13.6		2	14.3
Psychotic disorders (ICD-10 F2)	42	52.5		11	50.0		21	47.7		10	71.4
Affective disorders (ICD-10 F3)	13	16.3		4	18.2		8	18.2		1	7.1
Others	14	17.5		4	18.2		9	20.5		1	7.1
Psychosocial functioning (PSP)	3.74	1.75		4.80	2.78		3.72	1.61		3.22	1.72
Severity of illness (CGI)	5.00	1.00		4.55	1.21		5.11	0.78		5.00	1.36

### Qualitative Empirical Interviews

The qualitative subproject consisted of a semi-structured interview study with each of 15 patients, nurses, and psychiatrists. The interviews were conducted in the second hospital (facultative locked). We decided to conduct the interview study in this hospital, because the ward with the facultative locked setting allowed interviewees to compare an open with a locked setting directly. Therefore, an inclusion criterion for nurses and psychiatrists was to have work experiences on both open and locked acute psychiatric wards. Patients, on the other hand, were only included when they had experiences with involuntary commitment on the facultative locked ward. The sampling was conducted purposively to obtain a diverse sample selection. Further details regarding the sample characteristics and the procedures around the interviews are described elsewhere (12). The interviews were semi-structured and focused on different thematic aspects in the context of open-door policies. The data were analyzed following qualitative content analysis according to Mayring (46). After defining the relevant corpus of data, team members with different backgrounds (psychiatry, psychology, sociology, medical ethics) read and reread all interviews multiple times to gain familiarity with the data and identify and index potential themes and categories. Even though ward atmosphere and patient satisfaction were not directly addressed by the interview guideline, the former especially could be identified as a main theme in the interviews. During the following analysis, the coding evolved from concrete passages to more abstract levels, allowing coders to derive themes from the data directly while also complementing their analysis with a deductive approach. All statements which were made regarding the social climate or atmosphere on the ward were coded and assigned to the three subscales of the EssenCES (“Patients’ Cohesion,” “Therapeutic Hold,” or “Experienced Safety”) to produce comparable results between quantitative and qualitative data. The same was done for statements which referred to aspects comparable to the items of the ZUF-8. When analyzing the qualitative data, it was considered that some results might be caused by the special characteristics of a facultative locked setting. It was carefully selected and distinguished between statements which referred to an open or locked setting generally or could be transferred to a totally open or locked setting, and others which only seemed to be applicable to a facultative locked setting, as described above.

## Results

At the time of the assessment, neither the severity of illness nor psychosocial functioning of the patients differed significantly between the hospitals [CGI: *F*(2, 66) = 1.44, *p* = 0.245, PSP: *F*(2, 53) = 2.45, *p* = 0.096], and no significant correlations with any of the EssenCES subscales or the ZUF-8 could be observed.

**Table 3** gives an overview of the measures for ward atmosphere (EssenCES) and patient satisfaction (ZUF-8), which will, hereafter, be presented in more detail together with results from the qualitative subproject.

**Table 3:** Ward atmosphere (EssenCES) and patient satisfaction (ZUF-8) in total and per hospital.

	Total			Hospital 1 (locked)			Hospital 2 (facultative locked)			Hospital 3 (open)	
	***M***	***SD***		***M***	***SD***		***M***	***SD***		***M***	***SD***
Ward atmosphere (EssenCES)											
Patients’ Cohesion	11.52	4.05		11.78	5.08		11.48	3.57		11.21	3.89
Experienced Safety	11.23	5.27		9.17	6.00		11.05	4.96		15.21	1.93
Therapeutic Hold	13.65	4.93		11.26	5.88		13.89	4.36		16.86	2.57
Patient satisfaction (ZUF-8)	21.66	4.72		22.15	5.82		22.16	4.66		19.23	1.24

### Ward Atmosphere

**Figure 1:**
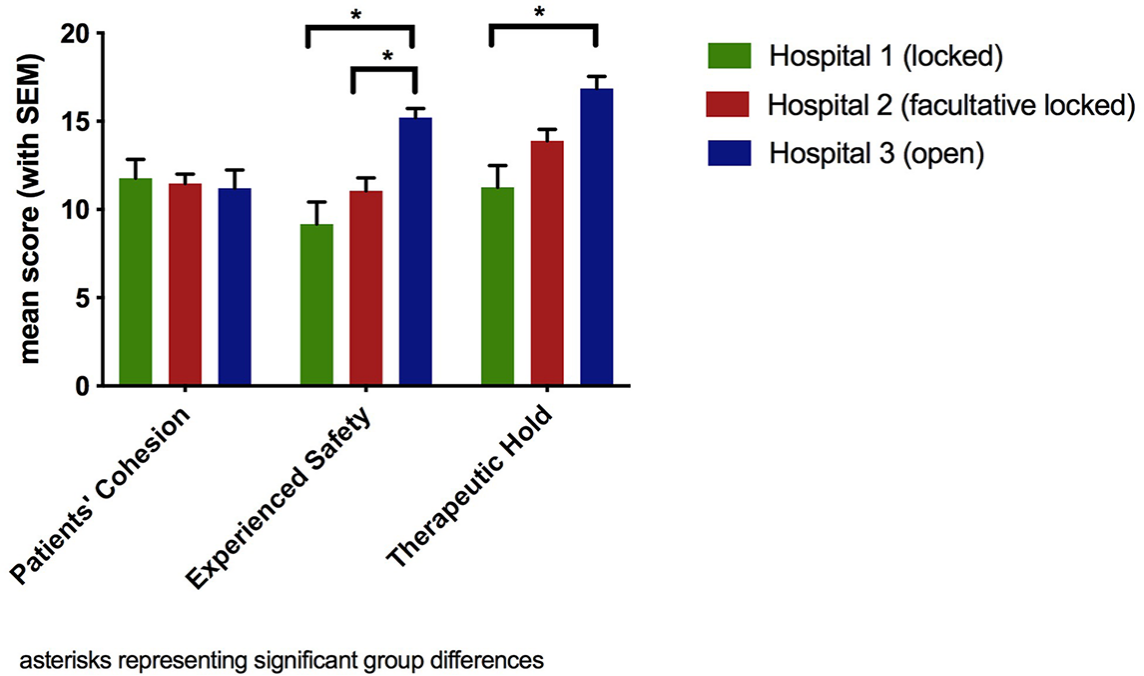
Subscales of ward atmosphere (EssenCES) per hospital.

The highest mean in the EssenCES in the total sample could be found for the subscale “Therapeutic Hold” with *M* = 13.65 (*SD* = 4.93), whereas the subscales “Patients’ Cohesion” (*M* = 11.52, *SD* = 4.05) and “Experienced Safety” (*M* = 11.23, *SD* = 5.27) were rated somewhat lower. **Figure 1** shows the results for all three subscales comparing the three different clinical settings. There were no differences regarding the subscale “Patients’ Cohesion,” which was confirmed by the statistical analysis (*F*(80) = 0.09, *p* = 0.915). A significant overall difference between the three hospitals was found for the other two subscales (“Experienced Safety”: *F*(80) = 6.58, *p* = 0.002; “Therapeutic Hold”: *F*(80) = 6.51, *p* = 0.002). Post hoc tests revealed that the open setting differed significantly regarding the subscale “Experienced Safety” both from the locked setting (*I-J* = 6.04, *p* = 0.002) and the facultative locked setting (*I-J* = 4.17, p = 0.022) and the open setting differed significantly from the locked setting regarding the subscale “Therapeutic Hold” (*I-J* = 5.59, *p* = 0.002).

The results of the semi-structured interviews with the patients, nurses, and psychiatrists are presented following the structure of the EssenCES and grouped into pro and contra arguments regarding the different settings.

#### Patients’ Cohesion

Concerning “Patients’ Cohesion,” the interviewees were mainly in favor of the open setting but also criticized facultative locked concepts.

##### Pro Open/Contra Locked

All groups agreed that open doors lead to a greater communicative exchange and support among patients.

*And then the understanding among the patients. There is much more interaction, much more communication about one’s own symptoms. Lovely, lovely groups form who sit together and who actually, in a way—once the therapeutic program is over—also structure the everyday life, which doesn’t exist when the door is simply locked. (Nurse 2)*

Furthermore, there are more possibilities for withdrawal and privacy for patients and nurses in the open setting, which, consequently, lowers the potential of conflicts among patients. This could lead to better relationships up to friendships which formed during the inpatient stay. All groups stated that, on the contrary, conflicts among patients occur more often in a locked setting than in an open setting due to the fact that the same patients are locked up together in a limited space where they cannot avoid each other.

*Well, simply that patients, loosely speaking, were threatening or bullying one another or whatever. And that’s something you definitely noticed. Well, especially during the time when the door had been locked for a while; that there was a certain tension in the air, so to speak. (Psychiatrist 9)*

Against the background that open-door policies often are associated with the allocation of tense or aggressive patients to different wards instead of one single intensive care unit, psychiatrists highlighted the benefits of having a heterogeneous group of more or less severely ill patients on one ward.

*They can go outside; they can go to another section. It mixes more with the healthier patients, too. In the past, you had all acute patients of the house in one section. It mixes more. (Psychiatrist 14)*

##### Contra Open/Pro Locked

Contrary to the psychiatrists, nurses stated that such a blending of patients may lead to a psychological strain for the less severely ill patients. Additionally, less acutely ill patients might experience a greater fear of more acutely ill patients and, therefore, be destabilized.

##### Contra Facultative Locked

Some answers in the interviews were directed specifically concerning the features of the facultative locked setting. Patients especially claimed that the intermittent locking of a ward in challenging situations creates an abrupt separation between patients, which, consequently, leads to further negative repercussions, such as feeling isolated or disadvantaged compared to the patients in an open setting. Additionally, all groups indicated that such a practice can lead to the apportionment of blame towards those patients who are considered responsible for the necessity of door locking.

#### Experienced Safety

Regarding “Experienced Safety,” interviewees expressed different opinions and arguments in favor of both the open and locked setting.

##### Pro Open/Contra Locked

A lower feeling of tension was reported by all groups in the open setting, leading to a lower potential of aggression and conflict, regardless of the specific patients who are currently on the respective ward.

*I’ve always experienced the locked door as a sort of pressure cooker, a kind of powder keg basically. The door was locked, and you noticed the tension in that section has intensified. That was the position in most cases anyway. (Nurse 11)*

According to the interviewees, this phenomenon can lead to a spiral of escalation, because patients who are already tense are made even more aggressive by the locked door and other patients become affected by this tension.

*You build up frustration not only as a reasonably healthy but also as an ill person. You can’t get out. The door is locked. And then frustration comes and … trash, littering, or demolishing walls or I don’t know. Frustration bubbles up because the door is locked. (Patient 1)*

Furthermore, all groups pointed out that there are always comparatively stable and balanced patients in locked settings who are “incarcerated” with potentially aggressive patients and, thereby, put at risk. This is in line with patients reporting that they experience less fear when the doors are open. Interestingly, nurses and psychiatrists both mentioned that a higher feeling of safety in a locked setting is often deceptive, because locking the doors cannot completely prevent aggression or serious incidents. Instead, they see the problem that mental health professionals can underestimate risk situations on a locked ward and do not pay enough attention to imminent dangers or crises.

*The second negative aspect of the locked door, I feel, is that a certain safety thinking sets in: “There’s nothing behind there anyway, nobody could do anything and all the dangerous things are gone now.” If someone then has a lighter or if someone tears off a chair leg in his psychotic force, as has happened, then one doesn’t have it in one’s head; that they can also tinker with stuff without something new getting in. (Psychiatrist 5)*

In this context, nurses and psychiatrists also discriminate between the safety experienced and real safety provided by locked doors.

*[ … ] that a certain risk is always at play and that you cannot necessarily minimize that risk by locking the door. And then the question—what really provides us with safety, what is perceived safety and how much true safety really exists with a locked door. (Nurse 7)*

This is also experienced by the patients who mention that mental health professionals do not react to aggressive behavior in the locked setting as consequently as in the open setting.

##### Contra Open/Pro Locked

As a negative effect of the open setting, the nurses and psychiatrists pointed out that watching the door makes nurses a target of aggression, whereas a locked door is often more accepted as a physical border (12).

Furthermore, patients and nurses stated that a locked ward or a locked division might enhance the feeling of safety for the remaining patients on the open wards or divisions as it separates them from more acutely ill and potentially aggressive patients.

*On the other hand, when the door was locked, when you have patients there who are antisocial, aggressive, whatever, then the patients of the other division of course experienced it as protection when the door was locked. (Nurse 11)*

##### Pro Facultative Locked

All groups reported some benefits of having the possibility of periodically locking a door. Patients and nurses expressed that some delusional patients might feel safer behind a locked door.

In addition, for nurses and psychiatrists, the door does not only represent safety for other patients but also for the mental health professionals themselves. It is highlighted that locking the door, at least for a short period, for example, when patients are in a crisis or in some cases of tense admissions, can lead to de-escalation and, therefore, makes the mental health professionals feel safer [see also (12)].

*And I don’t know if one should give up the possibility of once briefly locking the door. Simply to ensure safety. Not with the idea of leaving it like that, but like I said, to de-escalate the situation for a short time. (Psychiatrist 9)*

#### Therapeutic Hold

Similar to the opinion on “Patients’ Cohesion,” the interviewees mainly expressed the advantages of the open setting regarding “Therapeutic Hold.”

##### Pro Open/Contra Locked

The most important benefit of open doors regarding “Therapeutic Hold” was seen by all groups in the greater mutual respect between patients and mental health professionals, resulting in further positive effects for all groups.

*Well, it’s really like a liberation when you get out of there [the locked division]; you’re taken more seriously again, you feel. (Patient 14)*

The interviewees further mention that the greater respect and greater communication at eye level in the open setting leads to patients being more accessible, therefore, nurses and psychiatrists can build a relationship with them more easily. This is accompanied by more engagement of the mental health professionals and, therefore, more care in the open than in the locked setting. Patients also feel that the mental health professionals are more responsive and that there are more options to talk with someone when the doors are open.

*If I keep the ward open, I really need to establish a relationship with him. I have to, in order to get a bit of a promise that he won’t harm himself and I accomplish that much better when I enter this relationship openly, when I have to, and I depend on that when the door is open. (Psychiatrist 12)*

Additionally, all groups stated that the therapeutic relationship between patients and mental health professionals is strained in the locked setting, because patients might try to merely exploit mental health professionals to open the doors.

*Due to the locked door, you are exploited to merely function as a key in that situation, to open the door. Patients also don’t take you, in my opinion, they don’t take you therapeutically seriously anymore. You have this key and they only see that key and they try, for better or for worse, to get you to stick it in, turn, get to the other side and from there on, so to speak, react, act to leave the ward somehow. (Nurse 2)*

##### Contra Open/Pro Locked

In contrast to the benefits reported, nurses mentioned that time-consuming tasks in the open setting, such as watching the open door, binds resources which can, consequently, result in fewer mental health professionals available for patient contacts and less time for primary nursing (see also (12)).

### Patient Satisfaction

Concerning patient satisfaction, values ranged between *M* = 19.23 (*SD* = 1.24) and *M* = 22.16 (*SD* 4.66), and no significant difference could be found between the hospitals [ZUF-8: *F*(2, 73) = 2.14, *p* = 0.125]. The mean in the overall sample was *M* = 21.66 (*SD* = 4.27), which corresponds to 68% of the maximum value of 32. Patient satisfaction seemed to be relatively independent from the ward atmosphere; the measures of ZUF-8 correlated significantly only with the EssenCES subscale of “Therapeutic Hold” (*r* = 0.39, *p* = 0.001).

Statements from the qualitative interviews which could be interpreted as related to patient satisfaction and went beyond aspects of ward atmosphere referred mainly to the treatment offers within the hospital. All groups generally expressed points of criticism and suggestions of improvement concerning the treatment. Patients especially stated that the hospital did not offer sufficient psychotherapy or conversations with the staff in addition to pharmacological treatment and that there were too few opportunities for activities on the ward. These criticisms seemed to be more pronounced regarding the locked setting. However, the nurses and psychiatrists interviewed pointed out that the lower participation in therapies and activities was not primarily an effect of the door status but depended more on the patients’ current state of health. Most patients in the locked setting are acutely ill and in a mental condition in which they are less receptive to therapeutic offers. In this context, the interviewees gave the recommendation to adapt the therapeutic offers to the needs and capabilities of acutely ill and involuntarily committed patients.

## Discussion

### Ward Atmosphere

Considering the quantitative and qualitative data together, our results showed that ward atmosphere is associated with the ward setting.

(1) Regarding “Patients’ Cohesion,” the qualitative data indicated that an open ward setting can reduce conflicts among patients and, thereby, improve the mutual support. However, the quantitative data regarding this subscale exhibited relatively low values in all hospitals with no difference between the different settings. One possible explanation for this discrepancy might be that mainly more stable patients benefit from the factors mentioned in the interviews, such as distribution of tense patients and an increased radius of movement. The more severely ill involuntarily committed patients, on the other hand, might experience little support from their fellow patients in any case, due to symptoms, such as suspiciousness, or problematic behavior, such as aggression. Additionally, the qualitative results indicate that facultative locked settings might be especially problematic regarding patients’ cohesion, as abrupt changes in the door status can have a negative impact on the relationships among patients. Our result that the door status does not apparently have an impact on patients’ cohesion from the perspective of the patients themselves corresponds to the study of Schalast and Sieß (29). Even though Blaesi et al. (30) and Schalast and Sieß (29) indicated that there might be a positive influence of open doors on patients’ cohesion rated by mental health professionals, in line with Lo et al. (31) and our qualitative results, this does not seem to be very consistent or clear. We conclude from the overall data that patients do not apparently benefit from open doors regarding their cohesion to fellow patients.
(2) Considering “Experienced Safety,” our quantitative data revealed significantly higher values in the open compared to the facultative locked and locked setting. This is consistent with the results of previous studies (29–31), indicating a clear benefit of open doors for the experienced safety. Concordantly, the patients also emphasized the advantages of an open setting for their safety experience in our qualitative interviews. However, the mental health professionals did not only highlight the de-escalatory effects of open doors but also articulated that the possibility of door locking can increase the feeling of safety in specific situations.
(3) “Therapeutic Hold” was quantitatively rated significantly higher in the open setting than in the locked setting. This was clearly supported by the qualitative data. All groups consistently experienced the benefits of the open doors, producing mainly a greater mutual respect and a better therapeutic relationship between the patients and the mental health professionals. These results seem to contradict previous studies, which showed no improvement of the therapeutic hold on open wards (29–31). From our point of view, the variations in the quantitative measurements may result from the differences in the samples examined. While we focused on the views of involuntarily committed patients, Schalast and Sieß (29) defined no specific inclusion criteria for patients, and Blaesi et al. (30) and Lo et al. (31) limited their assessment to mental health professionals. The relevance of sample characteristics is further supported by the results of our qualitative interviews, indicating that involuntarily committed patients benefit primarily from open-door policies. According to our interviews, a reason for that might be that the dependent and instrumental relationship between patients and mental health professionals in the locked setting is at least partially changed to a therapeutic relationship at eye level in the open setting.

### Patient Satisfaction

Our qualitative data did not indicate a relationship between the open and the locked setting regarding patient’s satisfaction with the treatment. Similarly, our quantitative assessment of patient satisfaction with the ZUF-8 showed no differences between the three psychiatric inpatient settings examined. The overall value of patient satisfaction revealed that patients were neither clearly unsatisfied nor satisfied with the inpatient treatment experienced during their involuntary commitment. In the ZUF-8 validation on a sample of patients in a psychosomatic hospital, Schmidt et al. (42) reported a skewed distribution towards higher values with a mean of 26.3. In a sample of voluntary and involuntary patients who had just recently been admitted to a psychiatric hospital, Borbé et al. (47) reported a mean value of 24.7. Compared with this, our values assessed seem to be rather low. However, regarding the specific population of involuntarily committed patients, our results are consistent with those reported in other studies (38). Thus, our results support the previous findings that involuntarily committed patients experience lower satisfaction than voluntary patients (36–38). This impact of the patients’ legal status might also be a good explanation of the findings of other studies which—unlike our own study—showed a relationship between patient satisfaction and the door status (37, 39). In those studies, both voluntary and involuntary patients were assessed, which probably led to a confounding between the effect of the door status and the legal status, as involuntarily committed patients are usually treated on locked and voluntary patients on open wards. The fact that we assessed patient satisfaction on open vs. locked wards solely from the perspective of involuntarily committed patients could be an explanation why we did not find such a difference between the different settings.

### Relationship Between Ward Atmosphere and Patient Satisfaction

Regarding the relationship between ward atmosphere and patient satisfaction, the only correlation we found was with the subscale of “Therapeutic Hold.” As this statistical correlation was only small to moderate, there seems to be no strong relationship between the two scales. This is in line with previous studies (48, 49) and supports the assumption that ward atmosphere and patient satisfaction are rather independent from each other. On the other hand, the interviewees in the qualitative subproject saw a relationship between satisfaction with treatment and the amount and quality of therapeutic offers. Other studies also assume a link between patient satisfaction and therapy, among others showing satisfied patients are more adherent to the treatments offered (36). However, the direction of the relationship between patient satisfaction and “Therapeutic Hold” in our study remains unclear. On the one hand, more satisfied patients might have experienced greater therapeutic hold. On the other hand, a greater therapeutic hold might have improved patient satisfaction. It would be interesting to investigate this relationship further in the specific context of involuntarily committed patients in the future.

## Strengths and Limitations

The main strength of our study is its mixed methods design using both quantitative and qualitative approaches to evaluate ward atmosphere in psychiatric hospitals with different ward settings and door policies. A further strength is that we also assessed patient satisfaction using a standardized questionnaire, especially because we focused our quantitative study on involuntarily committed patients and, therefore, on those persons who are most severely affected by the respective door policies. We ensured that our results were not limited to the patients’ view by the addition of nurses and psychiatrists’ perspectives in the qualitative interviews. As we assessed all involuntarily committed patients in the respective hospitals, the quantitative comparison was not limited to single wards or to a selective subgroup of patients. However, we are aware that the comparison between different hospitals always entails the risk of other influencing factors (such as architecture) which cannot be ruled out completely.

A major limitation of our study is the low response rate in the quantitative assessment of patients in the locked setting. The low rate might be caused by limited resources in the respective hospital due to the large total sample of involuntarily committed patients during the period assessed. However, we checked that the subsample of this hospital did not differ from the total sample of participating patients regarding specific characteristics (such as gender, age, previous hospitalization, or diagnoses). The response rates in the other two hospitals were higher and comparable to other studies examining the specific group of involuntarily committed patients (50, 51). To rule out any systematical bias in patient recruiting in all participating hospitals, we compared the sample of patients who filled out the questionnaire with the whole sample regarding sociodemographic aspects and found no relevant differences.

A second major limitation is that not all quantitative and qualitative assessments were conducted in all groups (nurses, psychiatrists, and patients) and hospitals. This limits the comparability of our results with previous studies, especially regarding the mental health professionals who were not included in the quantitative assessment. Furthermore, it is possible that the ZUF-8 was not the most suitable instrument to assess patient satisfaction in the context of open-door policies, as some items, such as those which ask whether one would recommend the treatment in the hospital to others, do not seem applicable to the specific situation of involuntarily committed patients. However, the ZUF-8 was developed especially for the psychiatric inpatient setting (35) and is an established instrument used in previous studies which included patients under involuntary commitment (37). Concerning the qualitative interviews, the limitation is that they were conducted in a context where the facultative door locking affected a small division of one ward (instead of the facultative locking of the door for the whole ward). We tried to consider the influence of this characteristic in the analysis; however, it is possible that this specific aspect plays a role in some of the statements given.

## Conclusion

Involuntarily committed patients experience a better ward atmosphere regarding “Experienced Safety” and “Therapeutic Hold” in the open psychiatric setting when assessed by the EssenCES. Considering that open-door policies target mainly security aspects or the therapeutic relationship between patients and mental health professionals, this seems plausible. Regarding “Experienced Safety,” the qualitative data further revealed specific advantages of a facultative locked setting which go beyond those being observed in a completely open or locked setting, such as short-term de-escalation. Patient satisfaction assessed by the ZUF-8, however, was not specifically associated with ward atmosphere or door policy. Qualitative data also indicated that, except from the support received from the staff, door policies do not influence the satisfaction with psychiatric treatment for involuntarily committed patients. Though, considering the correlation between patient satisfaction and “Therapeutic Hold,” our results emphasize the need for an appropriate and more specific instrument to assess the patient satisfaction of those under involuntary commitment. With such an instrument, differences regarding patient satisfaction between psychiatric inpatient settings with different door policies might be found in future studies. Such studies should also address the research question whether and how patient satisfaction in the specific population of involuntarily committed patients can be improved as this could help to enhance different aspects of treatment (e.g., treatment adherence) and, thereby, help to reduce involuntary interventions.

Given the fact that involuntarily committed patients and—at least under specific circumstances—mental health professionals experience better therapeutic relationships and a greater feeling of safety in hospitals with an overall therapeutic and organizational setting which enables the (permanent) opening of ward doors, this can be regarded as an argument to further promote and evaluate open-door policies in psychiatric practice. Thereby, future studies should investigate (1) a possible moderator or mediator effect of ward atmosphere on objective security aspects, such as the use of coercive interventions or the likelihood of serious incidents, and (2) whether it is more advisable to maintain the option of intermittent door locking in specific situations or to forego such an option completely.

## Data Availability

Given the small sample sizes and the detailed reports in the qualitative subproject, making the data publicly available would compromise privacy and anonymity of the research participants. Furthermore, the quantitative and qualitative datasets generated contain data that have not been analyzed and published yet. Upon reasonable request, they might be available at a later time from the corresponding author.

## Ethics Statement

This study was carried out in accordance with the recommendations of the Research Ethics Committee of the Medical Faculty of the Ruhr University Bochum with written informed consent from all subjects. The protocol was approved by the Research Ethics Committee of the Medical Faculty of the Ruhr University Bochum (Reg. No. 15-5452).

## Author Contributions

IO, JV, GJ, and JG made substantial contributions to the conception and design of the qualitative subproject and GJ and JG to the conception and design of the quantitative subproject.JB, IO, and JG recruited and interviewed the participants in the qualitative subproject. The qualitative data were analyzed and interpreted by SE, JB, JK, IO, and JG. JK, BU, H-JA, SZ, PN, GJ, and JG managed the collection and preparation of the quantitative data, which were mainly analyzed and interpreted by SE and JG. SE, JB, and JG wrote the first draft of the manuscript. All authors read the manuscript and were involved in revising and finalizing it.

## Funding

The Medical Faculty of the Ruhr University Bochum supported this study financially by a grant for the Department of Psychiatry, Psychotherapy and Preventive Medicine (Head: Georg Juckel; Awardee: Jakov Gather; FoRUM-award Clinical Research, K093-15). We acknowledge support by the DFG Open Access Publication Funds of the Ruhr University Bochum.

## Conflict of Interest Statement

The authors declare that the research was conducted in the absence of any commercial or financial relationships that could be construed as a potential conflict of interest.
